# The agony and the efficacy: central mechanisms of GLP-1 induced adverse events and their mitigation by GIP

**DOI:** 10.3389/fendo.2025.1530985

**Published:** 2025-02-03

**Authors:** Jonathan D. Douros, Jonathan N. Flak, Patrick J. Knerr

**Affiliations:** ^1^ Indiana Biosciences Research Institute, Indianapolis, IN, United States; ^2^ Department of Pharmacology and Toxicology, Indiana University School of Medicine, Indianapolis, IN, United States

**Keywords:** GLP-1 - glucagon-like peptide-1, GIP - glucose-dependent insulinotropic peptide, obesity, incretin, pharmacology

## 1 Introduction

Incretin peptides are secreted from the intestine after nutrient ingestion and enhance glucose-stimulated insulin secretion (1). In healthy individuals glucagon-like peptide 1 (GLP-1) and glucose-dependent insulinotropic polypeptide (GIP) are the primary incretin factors (2, 3). However, GIP receptor (GIPR) activity is diminished in patients with type 2 diabetes (T2D), while GLP-1 receptor (GLP-1R) function remains intact (4). This finding, along with the obesogenic physiologic role of GIP (5, 6), motivated the development of GLP-1R agonists over GIPR agonists for the treatment of T2D (7–11). Long acting GLP-1 analogues were later approved for obesity based upon early observations that physiologic and pharmacologic GLP-1R agonism reduces food intake in preclinical models (12) and pioneering clinical trials of long acting GLP-1 analogues (13). Despite the substantial improvements in body weight and glucose control elicited by GLP-1R agonists, patient uptake and compliance are challenged by frequent gastrointestinal adverse events (GI AEs), including nausea and vomiting, that necessitate careful dose titration regimens to achieve efficacious exposure (14). Emerging preclinical and clinical evidence suggests that GIPR agonism can play a role in reducing the GI AEs of GLP-1R agonism and enable greater therapeutic potential.

## 2 Weight loss pharmacotherapies

### 2.1 GLP-1R and GIPR agonism reduce body weight in patients with obesity

Early GLP-1R agonists were approved to treat T2D (13, 15, 16), but the potential weight lowering efficacy for this class of molecules first seen in rodents was not confirmed in humans until clinical trials with the first long-acting GLP-1 analogue, liraglutide (12). Over 56 weeks, liraglutide (3 mg daily) induced 6.0% weight loss in patients with obesity and T2D (17) and 8.0% weight loss in patients with obesity alone (unless otherwise defined, weight loss is placebo controlled from baseline) (18). Further optimization of GLP-1 analogues to permit once-weekly time action led to the discovery of Fc protracted dulaglutide and the fatty diacid acylated semaglutide. Dulaglutide (1.5 mg weekly) produced middling weight reduction (3.1%) over 26 weeks in patients with obesity and T2D (13). However, semaglutide (2.4 mg weekly) is said to have “broken the sound barrier” (19), inducing 9.6% weight loss in patients with obesity and T2D (20) and 14.9% weight loss in patients with obesity alone (21) over 68 weeks of treatment. In fact, liraglutide (1.8 mg daily) and semaglutide (0.5 mg weekly) both significantly outperformed dulaglutide (1.5 mg weekly) for weight loss in head-to-head trials at doses that induce comparable HbA1c lowering (13, 22).

The mechanism underlying the impressive weight loss potential of GLP-1R agonists is primarily, if not exclusively, mediated by a reduction in energy intake. For instance, native GLP-1 infusion acutely reduced food intake in individuals without (23) or with T2D (24), and the short acting GLP-1R agonist exenatide exerted the same effect in patients with obesity (25). A similar effect was seen with chronic dosing of optimized, long acting GLP-1 analogues liraglutide and semaglutide in patients with obesity (26, 27) or T2D (28, 29). Clinical and preclinical data indicate that GLP-1R agonism does not impact energy expenditure or nutrient absorption/accretion over extended treatment (30). In fact, there is a well-documented counter-regulatory reduction in metabolic rate in both humans (31, 32) and rodents (30, 33) treated with GLP-1R agonists for extended periods due to the reduction food intake. Thus, all current evidence points to the suppression of energy intake being the underlying mechanism responsible for the beneficial effects of GLP-1R agonists on body weight in patients with obesity and/or T2D.

While GLP-1R agonism effectively reduces body weight in patients, there is a persistent need for greater efficacy of weight loss and comorbidity resolution. Unimolecular, multi-receptor agonists have emerged to meet this growing need. While numerous proof-of-concept candidate molecules have been disclosed (34–43), the only approved example of multi-receptor agonism for obesity and T2D is the dual GLP-1R/GIPR co-agonist tirzepatide (marketed as Mounjaro^®^ and Zepbound^®^) (34, 44). Tirzepatide (15 mg weekly) drives 15.7% weight loss over 72 weeks in patients with obesity and T2D (45) and 22.5% weight loss in patients with obesity alone (46). Receptor occupancy analysis demonstrates that 5 mg weekly tirzepatide engages the GLP-1R to a similar degree as 1 mg weekly semaglutide (47); at this dose, tirzeaptide drives ~8.3% weight loss compared to 6.6% for semaglutide over 40 weeks in the head-to-head SURPASS-2 trial in patients with obesity and T2D. Thus, tirzepatide appears to outperform semaglutide at doses that comparably engage the GLP-1R. This phenomenon has been widely attributed to two hypotheses that are not mutually exclusive. The first is that tirzepatide is a partial, biased GLP-1R agonist, a profile that confers superior glucose and weight lowering in preclinical models (47–51). Second, tirzepatide is a full, potent GIPR agonist. GIPR monoagonism drives weight loss and food intake reduction on its own in both humans (52) and rodents (53–55). Tirzepatide stimulates insulin secretion through the GIPR in human islets from healthy donors, supporting a role for GIPR agonism in its pharmacology (56). In further support of this *ex vivo* finding, the GIPR monoagonist LY3537021 reduced body weight by ~4.1% over 8 weeks in patients with T2D (n = 18) (57). It should be noted that GIPR antagonism paradoxically produces additive weight loss when paired to GLP-1R agonism in preclinical studies (58–60) and that this mechanism appears relevant in patients with obesity (61). This phenomenon has been discussed in detail elsewhere (62). While dual receptor co-agonism offers enhanced weight loss and metabolic benefits, it also appears to offer upside in mitigating GI AEs.

### 2.2 GIPR agonism serves to reduce GLP-1R mediated GI AEs in the clinic

Treatment with GLP-1R agonists can drive nausea, diarrhea, vomiting, and constipation, which often results in discontinuation of treatment (6-10% of patients) or reduction in dose (~15% of patients) (63). The most pronounced effects occur acutely upon treatment initiation and wane over the course of the first 90 days of exposure. This phenomenon is clearly observed in the STEP 2 trial of semaglutide (2.4 mg weekly), where a cumulative 33.7% of patients with obesity and T2D reported nausea throughout the duration of the trial (20). An elegant data analysis of the STEP-2 protocol shows the temporal dynamic of this effect, where the incidence of nausea grew from ~5% at the beginning of the dose escalation period (week 1; 0.25 mg weekly) to ~15% by the end of the dose escalation (week 13; 2.4 mg weekly). Over the next 56 weeks, patients reporting nausea steadily decreased to ~8% by the end of the trial. We will treat nausea as a proxy for GI AEs because similar patterns were reported for diarrhea, vomiting, and constipation across trials.

It has been hypothesized that the deleterious, nauseating effects of GLP-1R agonists and the beneficial, food-intake reducing effects are inextricably linked. However, this hypothesis is losing traction in light of emerging clinical data. In the SURPASS-2 trial, patients with obesity and T2D who received tirzeapatide (5 mg weekly) achieved greater HbA1c and body weight reductions than those receiving semaglutide (1 mg weekly) during the 40 week study (64) despite comparable putative GLP-1R occupancy at these doses (47). Interestingly, patients receiving tirzepatide report reduced overall GI AEs (40%), nausea (17.4%), diarrhea (13.2%) and vomiting (5.7%) compared to patients on semaglutide (43%, 19.2%, 13.7%, and 8.1% respectively). This is suggestive but not demonstrative that GIPR agonism reduces the GI AE incidence associated with GLP-1R agonism. Seminal studies by Knop et al. demonstrate that the addition of a long-acting GIPR agonist (LY3537021) significantly reduced the total number of GI AEs induced with liraglutide treatment by 16% and numerically reduced nausea by 15% and vomiting by 3%, but not diarrhea (65). It should be noted that, while the tolerability and efficacy profile of the GLP-1R agonist/GIPR antagonizing antibody maridebart cafraglutide warrants enthusiasm, it has not yet been directly compared to GLP-1R agonism or dual GLP-1R/GIPR agonism in the clinical setting. Collectively, these data demonstrate GIPR agonism can improve the tolerability profile of GLP-1R agonists in humans.

**Figure 1:**
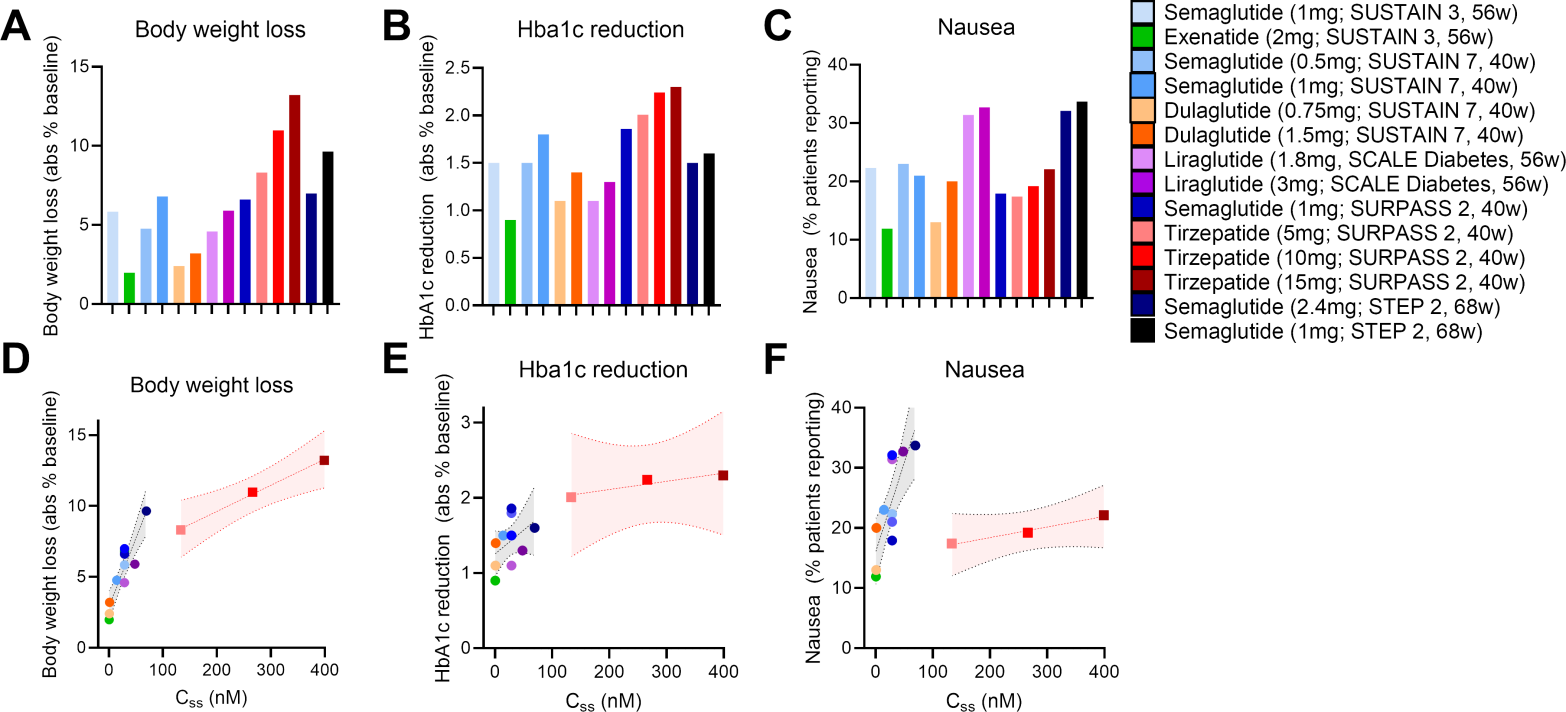
Tirzepatide exhits lower nausea compared to GLP-1R monoagonists across clinical trials. **(A)** Body weight loss, **(B)** HbA1c reduction, and **(C)** nausea reported across clinical trials as outlined. **(D)** Body weight loss, **(E)** HbA1c reduction, and **(F)** nausea as a function of the calculated circulating drug exposure (Concentration at steady state; C_ss_).

**Figure 2:**
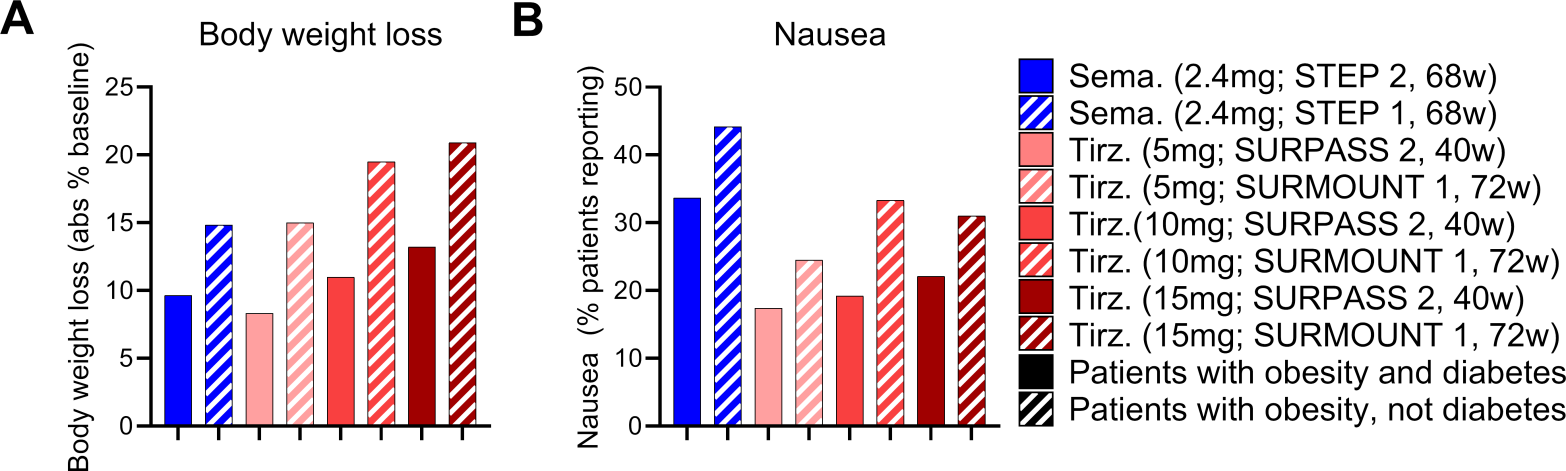
The effects of tirzeaptide and semaglutide are reduced in patients with obesity and diabetes compared to patients with obesity alone. **(A)** Body weight loss and **(B)** nausea reported across clinical trials as outlined. Groups in blue were treated with semaglutide while groups in red were treated with tirzepatide. Patients with obesity and T2D are shown in solid bars, while patients with obesity but not T2D are shown in the hatched bars.

With these points in mind, we undertook a broad assessment of published clinical data, curating the body weight loss (% from baseline), glycosylated hemoglobin levels (HbA1c), and nausea responses (% patients reporting) from the SUSTAIN 3, SUSTAIN 7, SCALE Diabetes, SURPASS 2, and STEP 2 trials (17, 22, 64, 66, 67) (**Figures 1A–C**). We assessed the GLP-1R agonists (exenatide, liraglutide, dulaglutide, semaglutide) and the dual GIPR/GLP-1R co-agonist tirzepatide as separate classes. We selected these trials due to their similarities in assessing patients with both obesity and T2D, treatment duration, clinical development phase (phase 3), and use of semaglutide as an active treatment arm. We also provide an assessment of body weight loss and nausea induced by semaglutide and tirzeapatide across patients with obesity and T2D in the STEP 2 and SURPASS 2 trials compared to those with obesity but not T2D in the STEP 1 and SURMOUNT 1 trials (**Figure 2**). All drugs followed a similar trend in which the calculated circulating concentration of the drug at stead state (C_ss_) exhibits significant positive association with body weight loss (**Figure 1D**). This relationship was not present for C_ss_ and HbA1c, potentially due to a plateau in glycemic control for both drug classes (**Figure 1E**). The GLP-1R monoagonists displayed a significant positive correlation between C_ss_ and nausea, which is not observed for the GIPR/GLP-1R dual-agonist (**Figure 1F**). Additionally, it is clear that tirzeaptide induces less nausea per nmol in circulation compared to the GLP-1R monoagonists despite the improvement in weight loss. In contrast, co-treatment of semaglutide with the amylin receptor agonist cagrilintide resulted in 29% of patients with obesity and T2D reporting nausea compared to 13% for cagrilintide alone and 16% for semaglutide alone (68). This analysis supports the hypothesis that nausea suppression by GIPR agonism (57, 65, 69) contributes to the better tolerability profile of tirzepatide (15 mg) at doses 6.25x higher than semaglutide (2.4 mg).

## 3 Mechanisms of action for GLP-1R agonists to control food intake

GLP-1R agonists primarily act to reduce body weight by suppressing energy intake. Preclinical studies show that both short acting agonists (i.e. exenatide) (50) and long acting agonists (i.e. liraglutide, semaglutide) (35, 37) acutely reduce food intake in rodents. Depending on the molecular properties (33), these agonists exert their effects by activating GLP-1R^+^ glutamatergic neuronal populations (70, 71), but not GABAergic neurons (72). It has been hypothesized that the effects of GLP-1R agonists to reduce energy intake are at least partially driven by nausea and other GI AEs. Under this model, GI AEs are an essential feature, not a bug, of the pharmacology. However, key preclinical studies in mice indicate otherwise. GLP-1R agonists reduce food intake by triggering both aversive or emetic neural and satiety signals which are dissociable in rodents (73–76). This finding opens the door for the development of weight lowering pharmacotherapies that suppress food intake without triggering nausea by targeting specific subsets of GLP-1R^+^ neurons. Tailoring a molecule to this specific purpose requires a substantial understanding of how the current class of GLP-1R agonists access and interact with the satiating and aversive neuronal receptor populations.

**Figure 3:**
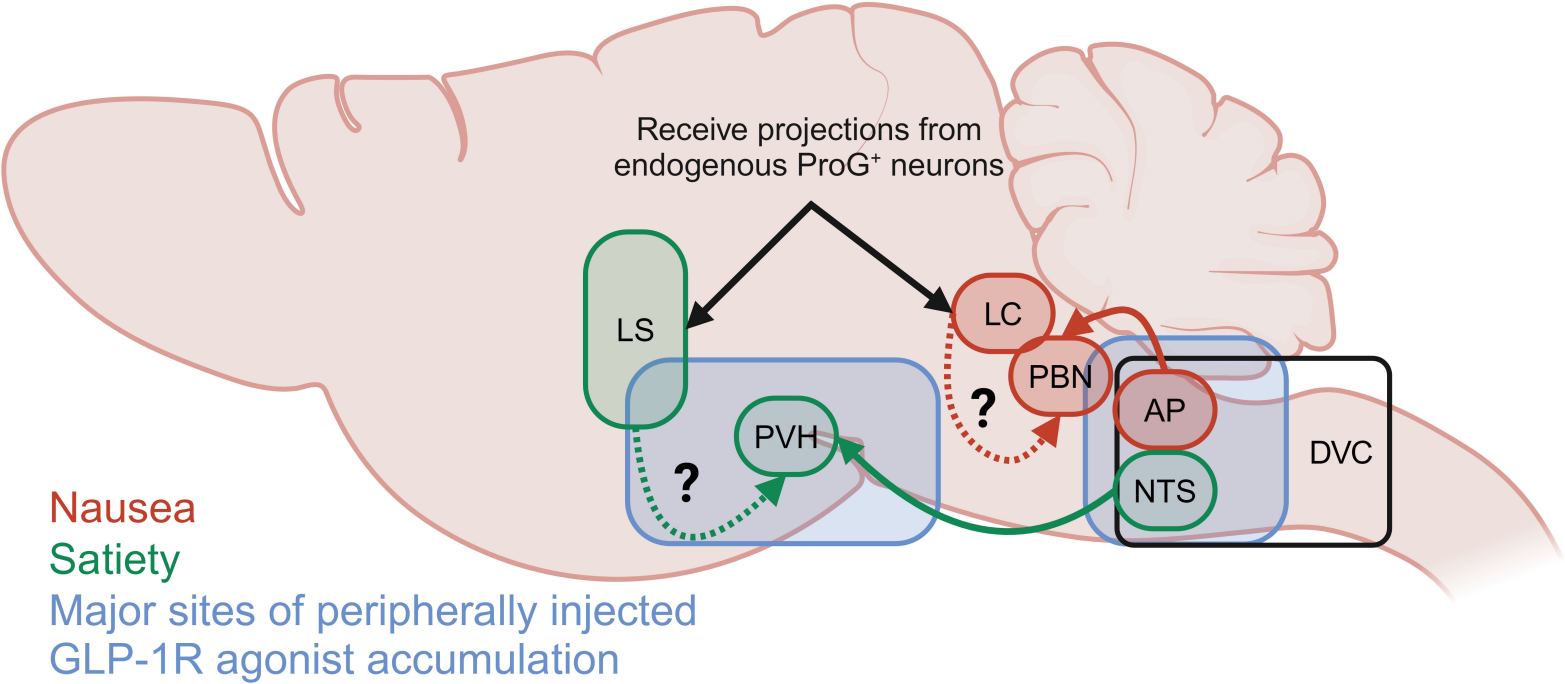
Summary of the primary brain depots for semaglutide as reported by Gabery et al. (33) and neuronal populations reported to mediate GLP-1R agonist-induced satiety (green) and food aversive, avoidant or nauseating behavior (red). Neuronal populations indicated by black arrows receive projections from pre-proglucagon positive (ProG^+^) neurons.

Acute treatment of diet-induced obese mice with labelled semaglutide has been shown to allow the drug to access circumventricular organs that lack a substantial blood brain barrier, including the area postrema (AP), median eminence (ME), vascular organ of the lamina terminalis (OV), and subfornical organ (SFO) (33). In addition, labelled semaglutide can also cross the blood brain barrier to the caudal lateral septal nucleus (LSc), septofimbrial nucleus (SF), arcuate nucleus (ARC), median preoptic nucleus (MnPO), dorsal vagal complex (DVC). At steady state, labelled semaglutide also appeared in the choroid plexus (CHPL), dorsomedial hypothalamic nucleus (DMH), medial mammillary nucleus (MM), paraventricular hypothalamus (PVH), supraoptic nucleus (SO), and tuberal nucleus (TU) (**Figure 3**). In rodents, intracerebroventricular (ICV) administration of the GLP-1R antagonist exendin-9 suppressed the effects of peripheral GLP-1R agonist administration (liraglutide and exenatide) on food intake reduction. Exendin-9 administered IP was unable to block this effect, indicating a dominant role for CNS GLP-1R populations (77). Indeed, CNS glutamatergic neuron receptor populations, but not GABAergic neurons, are necessary for liraglutide-induced food intake and body weight reduction (71, 72). Furthermore, studies using a peripherally administered exenatide analogue conjugated to vitamin B12, which is sterically hindered from accessing regions protected by the blood brain barrier, improves glucose control in comparable fashion to unmodified exenatide, presumably at the level of the β-cell, but does not induce emesis in the house musk shrew (*Suncus murinus)* or rats (78, 79). It should be noted that physiologic GLP-1R agonism in the periphery mediates food intake reduction via vagal afferent signaling as rats with a surgical subdiaphragmatic vagal deafferentation (SDA) exhibit a more mild food intake suppression in response to acute administration of GLP-1 analogues than sham operated animals (80, 81). Collectively, this indicates a role for CNS GLP-1R populations to mediate food intake suppression by pharmacologic, and to a lesser degree physiologic, GLP-1R agonism.

The specific effects of GLP-1R agonism on food avoidance, food aversion and emetic behaviors have been delineated more clearly in recent years. While mice and rats do not vomit, GLP-1R agonism induces emesis in the musk shrew and is associated with an increase in cFOS staining in the AP, nucleus of the solitary tract (NTS), DMH, VMH, lateral hypothalamus (LH), and PVH that is blocked by GLP-1R antagonism (77). Interestingly, daily peripheral exenatide administration induced a transient reduction in food intake, which regresses to baseline over time. This differs from the pattern of liraglutide, which induced an initial reduction in food intake with a transient increase in kaolin intake that both undergo partial tachyphylaxis over time (82). This comports with the human data showing that different GLP-1 analogues can have different food intake suppression and nausea profiles (82).

Location of the GLP-1R population plays an essential role in satiating versus aversive effects of GLP-1R agonists (**Figure 3**). Hypothalamic ARC or nodose ganglion (NG) GLP-1Rs are not necessary for the food intake reducing effects of GLP-1R agonists, while the hindbrain DVC is essential for this function (76). GLP-1R ^+^ neurons in the DVC, including the AP, are predominately (40-65%) responsive to aversive signals (LiCl, cinacalcet). Additionally, GLP-1R ^+^ neurons in the hindbrain locus coeruleus (LC) (83) partially mediate food avoidance behaviors of direct exenatide injection and peripherally injected semaglutide assessed by kaolin intake (84). These LC neurons receive projections from pre-proglucagon expressing neurons, indicating a possible role in the physiologic control of food intake by the endogenous GLP-1 system. It should be noted that the GLP-1R^+^ DVC/AP neurons project to the lateral parabrachial nucleus (PBN), which is in close proximity to the LC; thus, the PBN may serve an integrating function in the food aversive and avoidant effects of GLP-1R agonism (**Figure 3**).

On the other hand, GLP-1R^+^ neurons in the nucleus of the solitary tract (NTS) predominantly (~60-70%) respond to satiety signals and project to the PVH. Additionally, the forebrain lateral septum (LS) appears to mediate the non-avoidant food intake reducing effects of GLP-1Rs in that direct exenatide injection into the rat LS suppresses chow intake but does not enhance kaolin intake (85). Additionally, blockade of GLP-1Rs in the LS with exendin-9 alone enhanced sucrose and oil intake indicating a physiologic role for these neurons to suppress food intake. Interestingly, exenatide in the LS does not impair anxiety-like behaviors or operant responding, which are key functions for cells within this region that indicate a fine tuning of GLP-1R action in this region.

Finally, very few DVC neurons (~6-12%) are responsive to both aversive and satiating cells, indicating that there are indeed separate nausea and satiety effects by GLP-1R agonism. In further support of this notion, GLP-1R agonists can still reduce food intake when the aversive pathways of the AP are inhibited; ablation of the nausea-mediating AP, but not NTS, GLP-1R ^+^ neurons eliminates the conditioned taste avoidance response to semaglutide in rodents (70, 74). This finding is not unique to mice, as a similar response has been reported in musk shrews treated with direct injections of exenatide with and without exendin-9 into the AP and NTS (86). Thus, it appears possible to divorce nausea from satiation in mice treated with GLP-1R agonists by discovery of GLP-1R agonists that preferentially access and activate the NTS but not the AP.

In summary, long-acting GLP-1R agonists primarily reach the circumventricular organs of the hindbrain and food-intake controlling centers of the hypothalamus. In the hindbrain these agonists act to reduce food intake through both satiating and aversive actions. While it has been postulated that these two actions are inextricably linked more recent data shows they can indeed be divorced which opens the opportunity for tailored pharmacology that provide food intake reduction without the adverse events typically associated with GLP-1R agonism.

## 4 GI AEs associated with GIPR modulation

**Figure 4:**
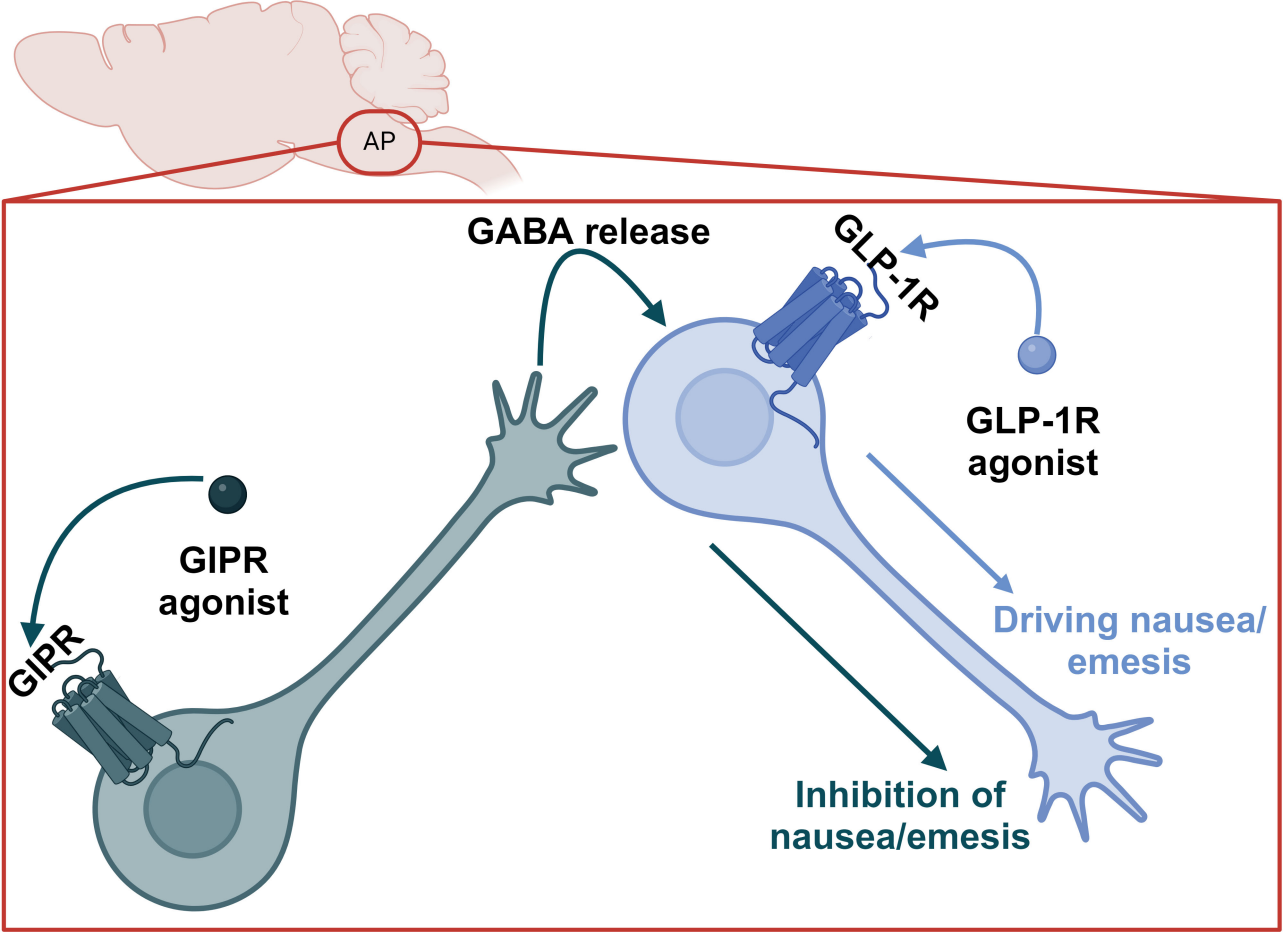
Proposed model for the suppression of GLP-1R agonist-induced nausea and emesis by GIPR agonism in the AP.

Because the food aversive and avoidant effects of GLP-1R agonism appear to be dissociable from satiety, it is theoretically possible to leverage this finding in a next generation, tailored pharmacology. Indeed, GIPR agonism can blunt the nauseating effects of GLP-1R agonists in humans (52). In preclinical models, GIPR agonism reduces food intake and body weight via CNS GABAergic neurons (53–55); this effect is additive with GLP-1R agonism and mediated by the same CNS GABAergic GIPR^+^ population (34, 54). In preclinical studies, GIPR/GLP-1R co-agonism produces less nauseating effects compared to GLP-1R agonism alone as measured by kaolin intake in mice and rats and emetic events in musk shrews (69). Critically, the reduction in nauseating or emetic responses induced by GIPR agonism does not limit the short term (72h) weight loss in any preclinical model (69). This supports the conclusions of Huang et al., that satiety (and by extension efficacy) and adverse events can be dissociated (76) and the clinical data outlined above. The precise mechanisms for this effect are unclear; however, intriguing hypotheses emerge from single cell RNA sequencing data in rodents. First, GIPR^+^ neurons in the rat hindbrain are located primarily in both inhibitory and excitatory neurons in addition to some expression in oligodendrocytes (69, 87, 88), while GLP-1R is primarily expressed in excitatory neurons. There is limited co-expression of GIPR and GLP-1R (88). It has also been demonstrated that inhibitory GABAergic CNS GIPR ^+^ neurons are necessary for the food intake reducing effects of GIPR agonists and dual incretin co-agonists (54, 55), while excitatory glutamatergic CNS GLP-1R ^+^ neurons are necessary for the food intake reducing effects of GLP-1R agonists (71). This suggests an inhibitory GABAergic signal from the GIPR agonists dampens GLP-1R-mediated aversive, avoidant, or emetic signals, which may be accomplished by unique projections of GIPR neurons to GLP-1R ^+^ neurons in the AP (**Figure 4**). Therefore, this circuit could curb nausea caused by GLP-1R agonism but not interfere with GLP-1 signaling in the NTS, which induces satiety. This model is also satisfying from an evolutionary biology perspective in which GLP-1 and GIP do not have redundant functions in the brain, but rather fit into complimentary niches. It is noteworthy that GIPR agonism can also attenuate PYY mediated nausea in preclinical models (89), suggesting optimism for either dual or triagonist analogues of PYY, GLP-1, and GIP.

## 5 Conclusions

Incretin receptor agonists including GLP-1R agonists and dual GLP-1R/GIPR co-agonists reduce food intake and drive weight loss in patients with obesity. These therapeutic interventions also drive a variety of GI AEs. Emerging data in clinical and preclinical studies suggests the adverse and efficacious effects of these pharmacotherapies can be dissociated, and that GIPR agonism improves the GI AE profile of GLP-1R and dual incretin receptor agonists. The preclinical data suggests the hypothesis that inhibitory GIPR ^+^ neurons act specifically on GLP-1R^+^ neurons that drive nausea and food aversion or avoidance to dampen GLP-1R mediated nausea without reducing efficacy, potentially lifting the ceiling of GLP-1R agonist-induced reductions in body weight.
